# Adoptive Immunotherapy in Postoperative Non-Small-Cell Lung Cancer: A Systematic Review and Meta-Analysis

**DOI:** 10.1371/journal.pone.0162630

**Published:** 2016-09-12

**Authors:** Yuan Zeng, Wenli Ruan, Jiaxi He, Jianrong Zhang, Wenhua Liang, Yaoqi Chen, Qihua He, Jianxing He

**Affiliations:** 1 Department of Cardiothoracic Surgery, the First Affiliated Hospital of Guangzhou Medical University, Guangzhou Institute of Respiratory Disease & China State Key Laboratory of Respiratory Disease, No 151, Yanjiang Rd, Guangzhou, 510120, Guangdong Province, PR China; 2 Guangzhou Zisheng Biotech Company Limited, No 11, Zhu Jiang East Rd, Guangzhou, 510120, Guangdong Province, PR China; Cardiff University, UNITED KINGDOM

## Abstract

**Background:**

Adoptive immunotherapy (AI) has been applied in the treatment of non-small-cell lung cancer (NSCLC) patients, but the value of postoperative AI has been inconclusive largely as a result of the small number of patients included in each study. We performed a systematic review and meta-analysis to address this issue for patients with postoperative NSCLC.

**Methods:**

Pubmed, Embase, Cochrane Library were searched for randomized controlled trials comparing adoptive immunotherapy with control therapies in postoperative NSCLC patients. The primary endpoint was overall survival. Hazard ratio (HR) was estimated and 95% confidence intervals (CI) were calculated using a fixed-effect model.

**Results:**

Compared with control therapies, analyses of 4 randomized controlled trials (472 patients) showed a significant benefit of adoptive immunotherapy on survival (hazard ratio [HR] 0.61, 95% CI 0.45–0.84, p = 0.002), and a 39% reduction in the relative risk of death (no evidence of a difference between trials; p = 0.16, I² = 42%). In subgroup analyses by treatment cycles and treatment regimen, significant OS benefit was found in combination therapy of AI with chemotherapy, regardless of whether or not the treatment cycles were more than 10 cycles.

**Conclusion:**

Adoptive immunotherapy has the potential to improve overall survival in postoperative NSCLC. The findings suggest this is a valid treatment option for these patients. Further randomized clinical trials are urgently needed.

## Introduction

Lung cancer is the most commonly diagnosed cancer worldwide. It is the leading cancer site in males, accounting for 17% of the total new carcinoma cases and 23% of the total carcinoma deaths. In females, it is the fourth most generally diagnosed cancer and the second leading cause of cancer death.[1] Non-small cell lung cancer (NSCLC) stands for about 85% of the lung cancer cases worldwide and the 5-year overall survival (OS) rate is close to 15%.[2] Surgery is thought the most beneficial therapy choice, but only 20–25% of tumors are suitable for potentially radical resection.[3]

Through cancer resection techniques have seen much improvement, little advancement has been made in the last 30 years in regards to distant recurrence and subsequent mortality,[4] which is unacceptably high, even for patients in early stages with no nodal or other metastatic involvement.[5] Platinum-based doublet chemotherapy, though claimed to improve the prognosis of patients with NSCLC after surgical resection, has limited impact on survival and has seemingly reached a plateau in the past decade.[6,7]

More recently, newly developed tumor immunotherapy techniques, adoptive immunotherapy (AI) in particular, have shown promising clinical benefits.[8,9] The treatment of patients with cell numbers that have been increased ex vivo is called adoptive cell transfer. Immunotherapy that is based on the adoptive transfer of naturally developing or gene-engineered T cells are able to mediate tumour regression in patients.[8] The types of adoptive immunotherapy are numerous; they include, but are not limited to, dendritic cells and Cytokine-induced killer (CIK) cells, Tumor-infiltrating lymphocytes (TILs), Lymphokine-activated killer (LAK) cells, Activated killer T cells and dendritic cells (AKT-DC), Natural killer T (NKT) cells, and γδ T cells.[10,11] However, the value of AI for postoperative NSCLC patients remains unclear because of their small size. Individual trials have not had sufficient statistical power to detect the moderate survival differences that might be expected of postoperative immunotherapy. A systematic review of all the available randomized evidence and the combination of the results of these trials in a meta-analysis might give sufficient statistical power to detect whether postoperative adoptive immunotherapy is beneficial or not in the treatment of NSCLC. We, therefore, carried out a systematic review and meta-analysis to provide more reliable and up-to-date evidence on the effect of postoperative AI in NSCLC patients through OS to identify the validity of postoperative AI.

## Methods

### Literature Search Strategy

PubMed, Cochrane Library and EMBASE databases were searched for randomized controlled trials that compared adoptive immunotherapy with no adjuvant treatment in NSCLC patients who had undergone surgical resection from the date of inception to January 2016. We used the following search terms: “cytokine induced killer”, DC-CIK, “tumor infiltrating lymphocytes”, “lymphokine activated killer”, “activated killer cells”, “gamma delta T cells”, “immunotherapy”, “Gene-modified T cells” with “non-small-cell lung cancer” or “NSCLC” and “randomized” in all fields. The primary outcome was the OS which was defined as the time between the date of randomization and death or the last date of follow-up.

### Selection Criteria

Only randomized controlled trials, studies involving NSCLC patients who had undergone operation, and studies comparing AI with a non AI adjuvant treatment were eligible for inclusion; non-curative resection cases were also included. Publications with no primary outcomes and retrospective or prospective observational cohort studies were excluded. All abstracts, presentations, conference, case reports, expert opinions, guidelines, and reviews were also excluded. When duplicated data were encountered, only the most complete and novel reports were included for data extraction and assessment.

### Data Collection and Study Quality

Data extraction was independently conducted by two reviewers (Yuan Zeng and Wenli Ruan) using a standardized approach. The following items were abstracted from the published articles: year of publication, study phase, the number of patients, operative method, clinical information on the study patients (age, sex, histology), overall survival and duration of followup. Discrepancies were resolved by consensus with a third author (Wenhua Liang). Two reviewers (Jiaxi He and Yuan Zeng) independently conducted the risk of bias assessment of the included studies using the Cochrane Collaboration’s tool.

## Statistical Analysis

We extracted the hazard ratios (HR) and the associated 95% confidence intervals (CI) for OS results to estimate treatment efficacy within the AI and control groups. If HR and CI were not mentioned, these were estimated where possible using the methods of Parmar.[12] The I-squared was calculated to quantify heterogeneity which was defined as low (25%–49%), moderate (50%–74%), or severe (>75%). Fixed-effect analysis model was used to calculate the HR. If the heterogeneity was severe, a random-effect analysis model would be used. In addition, significance of the HR was determined by the Z test, and defined as statistically significant when P < 0.05. A sensitivity analysis was also conducted to examine the impact on the overall results. Publication bias was assessed by a funnel plot. Cochrane Review Manager 5.2 was used for all analyses.

## Results

### Literature Search and Study Characteristics

**Fig 1:**
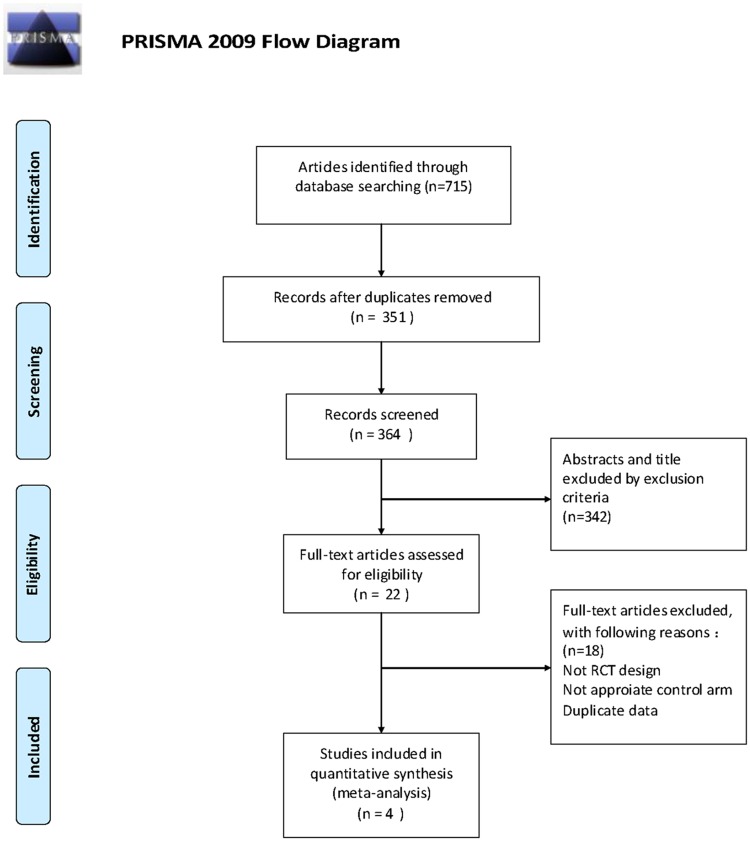
Selection and evaluation process of the eligible studies in the meta-analysis. *From*: Moher D, Liberati A, Tefclaff J, Altman DG, The PRISMA Group (2009). /deferred Reporting /terns for Systematic Reviews and Meta- Analyses: The PRISMA Statement. PLoS Med 6(7): e1000097. doi:10.1371/journal.pmed1000097 For more information, visit www.prisma-statement.org.

Pubmed, Embase, Cochrane Library revealed a total of 715 potential articles for analysis; of these articles, 364 were removed due to duplication. Careful screening of titles and abstracts revealed additional 342 ineligible studies. Twenty two full-text articles were selected for further investigation, of which four articles were finally selected for analysis.(Fig 1) The combined population of the 4 included studies was 472. The characteristics of the 4[11,13,14,15] studies are listed in Table 1. Among the 4 trials, three[13,14,15] trials received less than 10 cycles of AI adjuvant therapies, and one[11] received more than 10 cycles. In regard to the operative method, one[13] study performed only radical resection surgery; two[11,15] included both radical and non-radical; and one[14] included only non-radical resection surgery. In addition, three[11,13,14] of these trials compared AI with chemotherapy vs chemotherapy alone, while one[15] compared AI as monotherapy vs with or without chemotherapy.

**Table 1:** Characteristics of Included Studies for Meta-Analysis.

Study	Accrual Year	The number of patients	Operative method	Control	Treatment regimen	Treatment line	Follow-up period(years)	Stage
Kimura(2015)	2007–2012	101	Radical and non-radical resection	chemotherapy	AKT-DC + chemotherapy	12–14 cycles	5	IB- IV
Zhao(2014)	2010–2013	157	Radical resection	GP(gemcitabine plus platinum)	DC-CIK+GP	2 cycles	3	IIIa
Kimura(1997)	1986–1992	101	Non-radical resection	Either radiation therapy or chemotherapy	LAK and IL-2+ (either radiation therapy or chemotherapy)	At least 3 cycles	6	I -IV
Ratto(1996)	No report	113	Radical and non-radical resection	Chemotherapy + Radiotherapy or not	TIL and rIL-2+ Radiotherapy or not	1 cycle	3.3	II-IIIB

There was no significant difference in the number of patients, patients' age, gender, histology, and clinical stage between AI and control arms. All trials used resection as basic treatment before AI; One trial used AKT-DC as AI; one used DC-CIK; one used LAK plus IL-2; and one use TIL and rIL-2(recombinant interleukin-2). Some studies did not present the HR directly but the Kaplan-Meier curves. Therefore, the method reported by Parmar et al[12] was used to calculate and verify their HRs and 95% CIs. The characteristics of studies are shown in Tables 1 and 2.

**Table 2:** Characteristics of Included Studies for MetaAnalysis.

	Adoptive immunotherapy group (n = 234)	Control group(n = 238)
Trial		
Kimura(2015)	50	51
Zhao(2014)	79	78
Kimura(1997)	49	52
Ratto(1996)	56	57
Age		
Mean age	60.6	62.1
Sex		
Male	108	113
Female	70	68
Unknown	56	57
Histology		
Adenocarcinoma	141	133
Squamous	79	90
Large cell	2	3
Other	12	12
Clinical stage		
I	7	7
II	28	28
IIIA	132	135
IIIB	42	43
IV	25	25

**Fig 2:**
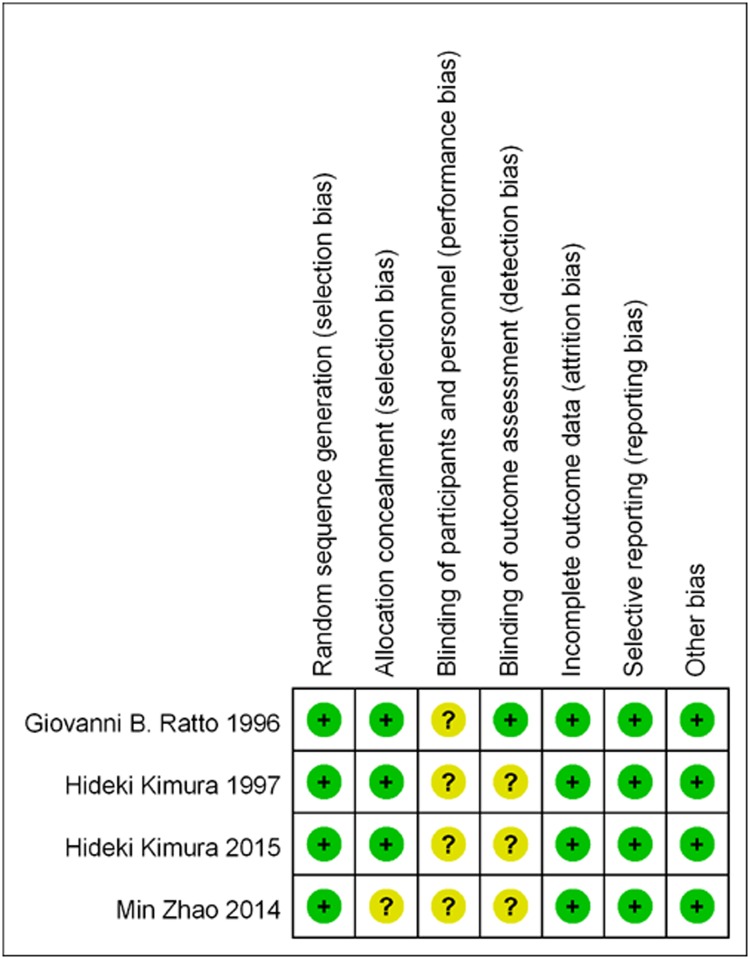
Risk of bias in included studies.

All four studies were randomized, and items were ranked as ‘‘low risk” based on the Cochrane Handbook. (Fig 2)

### Overall Survival

**Fig 3:**
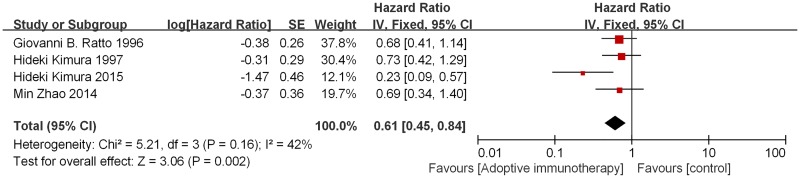
Forest plot of hazard ratio (HR) of overall survival in AI group versus control group.

**Fig 4:**
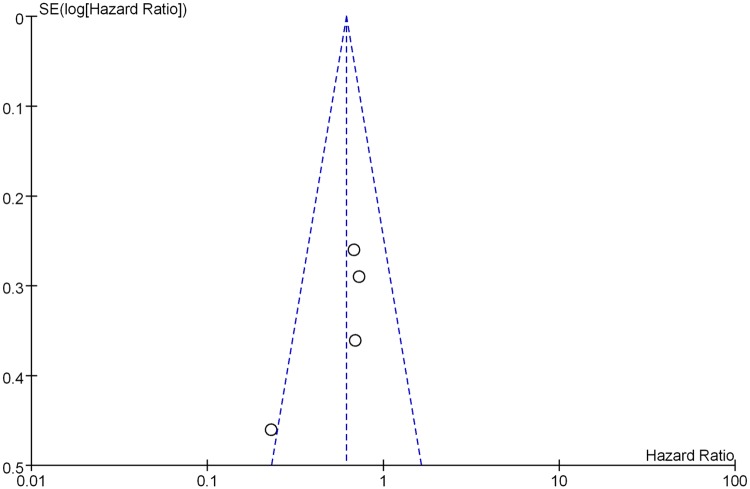
The total funnel plot of all groups.

The OS analysis demonstrated that AI significantly benefited patients’ survival compared with patients in the control group (HR 0.61, 95% CI 0.45–0.84, p = 0.002). This represents a 39% reduction in the relative risk of death. There was no evidence of significant heterogeneity among studies (p = 0.16, I² = 42%).(Fig 3) Sensitivity analysis, revealed consistent results. The total funnel plot can be seen in Fig 4.

### Subgroup Analysis

In a subgroup analyses by treatment line, three studies of 371 patients who received less than 10 cycles of AI adjuvant therapy had slightly better OS than those in the control group (HR 0.70, 95% CI 0.50–0.98, p = 0.04). No statistical heterogeneity was found for this outcome (I² = 0%; p = 0.98). Only one trial received more than 10 courses of AI adjuvant therapies, and it also had superiority in OS compared to the control group (HR 0.23, 95% CI 0.09–0.57, P = 0.001). In subgroup analysis based on treatment regiment, adoptive immunotherapy was found to be associated with significant OS improvement in the AI combined with chemotherapy group (HR 0.57, 95% CI 0.39–0.85). Moderate statistical heterogeneity was found for this outcomes (I² = 59%; p = 0.006). Only one trial compared with or without chemotherapy and also showed no statistically significant (HR 0.68, 95% CI 0.41–1.14). (Table 3).

**Table 3:** Results of subgroup analysis according to treatment line, treatment regimens for Meta-Analysis.

Stratified analysis		No. of studies	Cases (AI /Control)	Heterogeneity		Pooled HR (95% CI)	Text for overall effect	
				I^2^ (%)	P value		Z	P value
Treatment cycles	Less than 10 cycles	3	184/187	0.0	0.98	0.70 (0.50–0.98)	2.07	0.04
	More than 10 cycles	1	50/51	-	-	0.23 (0.09–0.57)	3.20	0.001
Treatment regiment	Combining with chemotherapy	3	178/181	59	0.08	0.57 (0.39–0.58)	2.73	0.006
	Comparing with or without chemotherapy	1	56/57	-	-	0.68 (0.41–1.14)	1.46	0.14

### Adverse Effects (AEs)

Three[11,14,15] of the four trials reported adverse effects. However, two of these trials did not provide the exact numbers of AEs. Among them, temporary fever, chills, and shivering were the main AEs identified. One trial reported no observed adverse reactions other than chills or fever, and the incidences were 37.25% and 35.3%.[11] What’s more, in one trial all of the patients experienced fever, chills, malaise, and nausea because of rIL-2 administration, but there were no Grade IV toxic events.[15]

## Discussion

To our knowledge, this study is the first meta-analysis to investigate the clinical efficacy of AI in postoperative NSCLC. Based on data from 4 randomised trials, our analysis confirms that adoptive immunotherapy has the potential to improve overall survival for patients with NSCLC after surgical resection.

As the immune system plays a vital role in surveillance and elimination of tumor cells, adoptive immunotherapy based on the adoptive transfer of naturally occurring or gene-engineered T cells can mediate tumour regression in patients with metastatic cancer.[8] AI has been applied in the clinic for several decades and has proven to be viable, less-toxic and effective in some patients, especially hepatocellular carcinoma,[16] melanoma,[17] leukemia,[18] and renal cell carcinoma[19]. In a randomized controlled trial in the 1990s, AI that used vivo-activated T cells showed clinical benefit in terms of prolongation of relapse-free survival for patients with hepatocellular carcinoma after resecting the primary tumor.[9]

Recently several randomized controlled trials[11,13,14,15,20,21] have indicated better survival with adoptive immunotherapy compared with chemotherapy or erlotinib alone in patients with NSCLC. Furthermore, some meta-analyses have indicated that progression-free survival and OS are improved in patients with advanced non-small-cell lung cancer, when compared with chemotherapy alone. However, the value of postoperative AI was inconclusive largely because of the small number of patients. In addition, AI is currently not a routine treatment for NSCLC. Therefore, a systematic review of all the available randomized evidence and the combination of the results of these trials in a meta-analysis might give sufficient statistical power for a clear decision on whether postoperative adoptive immunotherapy is beneficial in the treatment of NSCLC.

Most of the deaths seen after resection of NSCLC were due to remote recurrence.[5] This is most likely due to microscopic lesions that cannot be eliminated during surgery or metastatic disease undetected during resection.[22] Early animal experiments also showed that immunotherapy was effective in eliminating pulmonary metastases.[23] What’s more, a study show that AI decreased post surgical recurrence of hepatocellular carcinoma with hypothesis that immunotherapy may be most beneficial to patients with minimum residual tumours.[9] There are three trials including non-radical resection surgery in our study, so AI could probably be more effective for these patients. Possible explanation is that AI can eliminate these residual cancer cells and destroy the proliferating cancer cells so that it could decrease recurrence rate and improve OS for postoperative NSCLC. However, only one trial can not be able to make a clear decision whether adoptive immunotherapy is beneficial or not in radical surgery for NSCLC, so more research on radical surgery will be needed in the future. Although it is not a curative therapy for lung cancer, AI can efficiently therapy residual tumor cells after surgery in early stage NSCLC, and maintains patients in stable condition for the advanced stages of lung cancer.[10]

A large number of clinical trials suggested that there were neither serious adverse events (AEs) nor deaths caused by adoptive immunotherapy.[11,14,21,24] Fever and shaking chills were the only serious side effects observed in patients receiving adoptive immunotherapy. As a matter of fact, immunotherapy may alleviate some of the symptoms: patients had enhanced appetite, fatigue relief and better quality of life.[21] Hence, AI may also improve the quality of life of postoperative patients. However, even though three of the four trials reported treatment-related AEs, only one trial provided the exact numbers of patients, so we were unable to merge data for analysis.

Subgroup analyses were performed based on treatment cycles and treatment regimen. In the first subgroup based on treatment cycles, adoptive immunotherapy had slightly better overall survival than the control group in less than 10 cycles of AI, and no heterogeneity was found. One trail received more than 10 cycles of adoptive immunotherapy has significantly better overall survival revealed that the effect is likely different with different treatment cycles. In addition, Li et al.[25] found that the efficacy was significantly better in patients receiving CIK treatment for more than seven times than those receiving the treatment for fewer times. So multi-cycles can probably be recommended in patients with NSCLC.

As for the treatment regimen, a positive effect of AI for OS was observed only in combining with chemotherapy treatment, but not in comparing with or without chemotherapy treatment. So we confirm that additional AI is better than without AI, but data are not sufficient for any finite conclusions for comparing with or without chemotherapy treatment because of only one study performed the OS analysis, so randomized controlled trials are needed to solve this problem in the future.

There are a number of limitations in our study. Firstly, we were not able to analyze the recurrence data or the disease free survival and side effects. Secondly, we were incapable of providing the OS Kaplan–Meier curves of all included patients because of the lack of individual data. Thirdly, several of the HRs were not directly reported in the texts, therefore HRs were calculated using Engauge Digitizer. What’s more, non-curative resection cases were also included in our study. Finally, our analysis was based on published data from 4 RCTs with different AI protocols including CIK, AKT, TILs and LAK, so the clinical effect of AI on cancer may be influenced by variable cell dosage and phenotype of infusion products. The reliability of this systematic review and meta-analysis might be influenced by these limitations and the outcomes have to be interpreted with caution. Despite these limitations, there was no significant heterogeneity in the project, and it provided an important opportunity to advance to improve survival for NSCLC after surgical resection. This approach is particularly useful in merging these studies reported on a rather small number of patients. Therefore, we decided to get rid of these limitations from the analysis rather than limit the scope of our study.

## Conclusion

Based on data from 4 randomised trials, we confirm that AI has the potential to improve overall survival in postoperative NSCLC. There was no clear evidence of a difference in the effect on overall survival by treatment cycles. Subgroup analysis also revealed significant OS benefit in combination therapy of AI with chemotherapy. Though a number of limitations exist in our study, our findings provide greater incintive to study and include AI for postoperative treatment of NSCLC. Future large multicenter, randomized controlled trials, with suitable treatment protocols should be conducted to evaluate the safety and efficacy of AI.

## Supporting Information

S1 Table PRISMA 2009 Checklist. (DOC)
